# Multiple Sclerosis and Autoimmunity: A Veiled Relationship

**DOI:** 10.7759/cureus.24294

**Published:** 2022-04-19

**Authors:** Zineb Barkhane, Jalal Elmadi, Lakshmi Satish Kumar, Lakshmi Sree Pugalenthi, Mahlika Ahmad, Sanjana Reddy

**Affiliations:** 1 Research, Universite Hassan II Faculté de Médecine et de Pharmacie de Casablanca, Casablanca, MAR; 2 Facultad de Ciencias Médicas, Universidad Nacional Autónoma de Honduras, Tegucigalpa, HND; 3 Internal Medicine, University of Perpetual Help System, Daisy Antonio Laperal Tamayo (DALTA), Manila, PHL; 4 Internal Medicine, Bicol Christian College of Medicine, Legazpi, PHL; 5 Research, Ziauddin University, Karachi, PAK; 6 Medicine, Bogomolets National Medical University, Kiev, UKR

**Keywords:** common etiology, autoimmunity, demyelinating disease, autoimmune diseases, multiple sclerosis

## Abstract

Multiple sclerosis (MS) is an autoimmune inflammatory illness that affects the central nervous system (CNS) when the body's immune system attacks its tissue. It is characterized by demyelination and varying degrees of axonal loss. This article has compiled various studies elaborating MS and other autoimmune diseases (ADs) co-occurrence. Several conditions that fall into this category, including type 1 diabetes (T1D), rheumatoid arthritis (RA), Guillain-Barre syndrome (GBS), myasthenia gravis (MG), and many others, are found in MS patients and their relatives, suggesting one or more common etiologic mechanisms, including genetic, environmental, and immunological factors, supporting the concept of a possible influence of poly-autoimmunity on MS and the rest of ADs, as well as providing a significant feature for early detection of the disease and also a potential treatment option by clinical neurologists.

## Introduction and background

Multiple sclerosis (MS) is an autoimmune inflammatory disorder of the central nervous system (CNS) characterized by demyelination and variable degrees of axonal loss. Jean-Martin Charcot (1825-1893), a French neurologist, was the first to define MS as a distinct disease in 1868 [1]. After summarizing earlier studies, Charcot named the disease "sclérose en plaques" and added his own clinical and pathological observations. Around the world, 2.8 million people suffer from MS. According to a recent epidemiological study led by the National MS Society (NMSS), almost 1 million people are living with MS in the USA (913,925) [2]. Young individuals between the ages of 20 and 40 are the most affected, and it has a strong gender preference, with females being impacted two to three times more often than males [3]. The condition appears to be more prevalent in the northern hemisphere, with some genetic susceptibility in Scandinavian or North European-origin people [4]. Although the cause of MS is unknown, but it is thought that a complex polygenic background and environmental triggers, like vitamin D and Epstein-Barr virus (EBV), contribute to the disease phenotype through unknown gene-environment interactions [5]. 

The course of the disease differs among individual patients, ranging from a mild or even asymptomatic illness to severe disability. As a way of predicting prognosis and responsiveness to therapy, MS has been categorized into four clinical subtypes: relapsing-remitting (RR) (85% of MS cases), primary progressive (10%), progressive relapsing (5%), and secondary progressive (develops in 50-80% of RR patients) [6]. MS is characterized clinically by discrete episodes of neurologic impairment (referred to as "attacks" or "relapses"). The symptoms that these episodes cause differ significantly from patient to patient and are dependent on the location of the neurologic involvement. Common symptoms include numbness, tingling, weakness, eyesight loss, gait impairment, incoordination, imbalance, and bladder dysfunction. Patients are neurologically stable in between episodes, at least throughout the condition's RR phase [7]. However, residual symptoms may persist, and many patients report fatigue or heat sensitivity during the time between attacks.

MS is diagnosed when evidence of inflammatory-demyelinating damage in the CNS is disseminated in both time and space. The clinical history, neurologic examination, magnetic resonance imaging (MRI), and the exclusion of other diagnostic options are used to make the diagnosis. Other "paraclinical" procedures, such as cerebrospinal fluid (CSF) examination, evoked potential recording, urodynamic tests of bladder function, and ocular coherence tomography (OCT), may help establish the diagnosis for particular patients, but they are frequently unneeded [8]. Several advances in the treatment of MS have been made in recent decades. Symptomatic care focuses on alleviating specific symptoms such as fatigue, stiffness, bladder dysfunction, and pain, while immunomodulatory medicines help alter the course of the disease. Corticosteroids (methylprednisolone) and adrenocorticotropic hormone (ACTH) are anti-inflammatory and immunomodulatory hormones commonly used to treat acute relapses and speed recovery [9]. MS is hypothesized to be an immune-mediated disease in which the body's immune system affects the brain and spinal cord. Although no particular antigens have been found, most MS experts believe MS is an autoimmune disease (AD). Autoimmune comorbidities frequently occur in MS, which may arise due to genetic susceptibility to autoimmunity and overlapping pathological mechanisms common to several autoimmune conditions. This article aims to underline the co-occurrence of MS and other ADs by highlighting the pathophysiological similarities that may help clarify causality and aid the appropriate management of ADs.

## Review

Multiple sclerosis and type 1 diabetes

MS and type 1 diabetes (T1D) are chronic disorders caused by immune system dysregulation. Despite disparities in the organ systems targeted and the age of onset for each disease, both conditions share genetic, immunological, and environmental factors. The familial aggregation and higher susceptibility to both diseases in first-degree relatives than in the general population support a genetic predisposition [5,10]. 

Even though both diseases are linked to the human leukocyte antigen (HLA), the haplotypes differ depending on the condition [5,10]. The co-occurrence of MS and T1D has been considered unlikely [11], given that the HLA haplotype DRB1*1501-DQA1*0102-B1*0602 increases MS's susceptibility while protecting against T1D [11,12]. However, Marrosu et al. conducted a cohort study in 2002 in Italy, Sardinia to assess the prevalence of T1D in 1,090 people with MS and their parents and siblings and found an increased prevalence of T1D within MS patients of Sardinia and their first-degree relatives. Diabetes was three-fold and five-fold more common in patients with MS than in their healthy siblings (p = 0001) and the general population (p<0.0001) [13]. The presence of other relatives with MS enhanced the incidence of T1D in healthy siblings of MS patients (OR = 341; p = 0019) [13]. Patients with relatives having MS had a six-fold greater incidence of diabetes than healthy siblings of MS patients who did not have other relatives with the disease (p = 0001) [13] (Table 1). In Sardinia, MS is linked to DRB1*0405-DQA1*0501-DQB1*0301 and DRB1*0301-DQA1*0501-DQB1*0201, rather than DRB1*15-DQA1*0102-DQB1*0602, as it is elsewhere [14].

**Figure 1:**
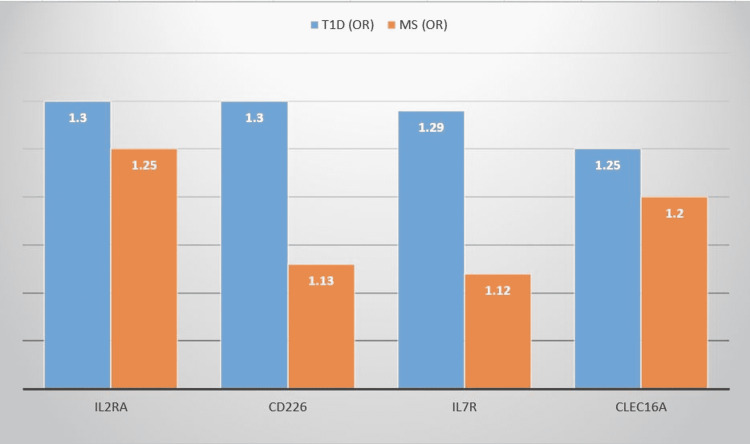
Non-HLA susceptibility loci for T1D and MS. OR: odds ratio; HLA: human leukocyte antigen; T1D: type 1 diabetes; MS: multiple sclerosis; IL2RA: interleukin-2 receptor subunit alpha; CD226: cluster of differentiation 226; IL7R: interleukin-7 receptor; CLEC16A: C-type lectin domain containing 16A.

T1D and MS share a few genes that have minor effects in common, according to genome-wide association studies (GWAS) [15,16]. These genes include interleukin-2 receptor subunit alpha (IL2RA), interleukin-7 receptor (IL7R), C-type lectin domain containing 16A (CLEC16A), and the cluster of differentiation 226 (CD226) [15,17], all of which have immunological functions (Figure 1).

Bechtold et al. conducted a cohort study in 2014 in Germany and Austria on a diabetic pediatric and adolescent population [18]. From January 1995 to October 2012, data from 56,653 patients with T1D were collected in 248 centers. The relative risk (RR) for MS in T1D was estimated at 3.35 to 4.79 (95% confidence interval (CI): 1.56 to 7.21 and 2.01 to 11.39), respectively. The study demonstrated a higher risk of co-occurrence of MS in pediatric and adolescent diabetic patients (Table 1). Furthermore, a Danish population-based cohort study conducted by Nielsen et al. in 2006 revealed that T1D patients were more than three times more likely to develop MS (RR: 3.26; 95% CI: 1.80-5.88; n = 11) [19]. In addition, first-degree relatives of MS patients had a 63% higher risk of developing T1D (RR: 1.63; 95% CI: 1.26-2.12; n = 56) [19] (Table 1). Also, Dorman et al. conducted a study in 2003 in the USA on female adults with diabetes manifestation before the age of 21 and their relatives. Data were collected for a familial autoimmune and diabetes (FAD) study that reported MS in 2% of women with T1D and 0.5% of their sisters; documented prevalence rates for MS in the USA extended from 0.06 to 0.17% (0.1% on average) for women [20]. As a result, a 20-fold increase in the prevalence of MS in T1D female adults was identified (p = 0.003) [20]. Non-diabetic sisters had a five-fold higher MS risk than the general population [20] (Table 1).

**Table 1:** Summary of included studies linking multiple sclerosis and type 1 diabetes mellitus. RR: relative risk; MS: multiple sclerosis; T1D: type 1 diabetes; CI: confidence interval.

References	Design	Data collected	Number of cases	Population	Results	Conclusion
Betchold et al. (2014) [18]	Cohort study	1995-2012	56,653 diabetics in 248 centers	Diabetic pediatric and adolescent population in Germany and Austria	RR for MS in T1D: 3.35 to 4.79 (99% CI: 1.56 to 7.21 and 2.01 to 11.39)	High-risk occurrence of MS in pediatric and adolescent diabetic patients
Nielsen et al. (2006) [19]	Cohort study	Two population-based disease registers: patients with T1D and patients with MS	Diabetics: 6,078, MS: 11,862, first-degree relatives of MS patients: 14,771	Diabetic patients diagnosed before age 20 in Denmark	More than the three-fold increased risk for MS in T1D; RR 3.26 (95% CI: 1.80-5.88) 63% increased risk for T1D in first-degree relatives of MS; RR 1.63 (95% CI: 1.26-2.12)	An intra-individual to a lesser degree an intra-familial co-occurrence of MS and T1D
Dorman et al. (2003) [20]				Adult diabetics in USA	Prevalence of MS: 0.06-0.17% (~0.1%) female diabetic adults: 20-fold increased risk of MS. Non-diabetic sisters: five-fold increased risk of MS	Increased prevalence of MS in female adults with T1D and their first relatives
Marrosu et al. (2002) [13]	Cohort study	Inpatients and outpatients with MS in a clinic	MS patients: 1,090, parents of MS patients: 2,180, siblings of MS patients: 3,300	Population in Sardinia, Italy	MS: increased risk of diabetes three-fold to five-fold. Relatives of MS patients: increased risk of diabetes six-fold	Increased risk of diabetes in MS patients and their relatives

T-cell-mediated autoimmunity is present in both MS and T1D, with the B-cell playing an important role. T1D is caused by T lymphocytes attacking pancreatic beta (β) cells once activated by particular insulin epitopes on antigen-presenting cells (APCs). Almost every child with two or more insulin antibodies (IAA, glutamic acid decarboxylase antibodies (GADA), IA-2A, ZnT8A) will eventually develop clinical T1D [21]. MS is a disease that develops in the CNS due to abnormal peripheral T-cell activation following antigen presentation. Antigens associated with myelin, such as myelin basic protein and myelin oligodendrocyte glycoprotein, are thought to contribute to the pathogenesis of the disease [22]. On the other hand, oligoclonal bands are immunological markers that can be detected with great sensitivity in over 85% of MS patients [23]. The T-cell response appears to be less disease-specific than one might predict for two very distinct diseases. In vitro, T-cells from T1D patients reacted to pancreatic islet and CNS antigens [24,25]. T-cells that target islet and CNS antigens are also found in the peripheral blood of MS patients [24].

The co-occurrence of MS and T1D is only partially explained by genetic variability, implying that other variables are at play [11]. The etiology of MS and T1D is hypothesized to be influenced by environmental variables. The increased prevalence of both diseases distant from the equator [26] suggests that vitamin D may play a role [27,28]. Vitamin D supplementation reduces the incidence of MS by a significant amount (OR for 400 units of vitamin D 0.60; 95% CI: 0.39-0.92), according to population-wide previous studies in MS [29,30]. Comparable research found a significant reduction in the risk of T1D later in life when vitamin D supplementation was started early in life (OR: 0.12; 95% CI: 0.03-0.51) [31], which was confirmed by a meta-analysis of prospective cohort trials [32]. In general, the proof supporting vitamin D's role in MS and T1D prevention is clear. Prior EBV infection is almost a common finding in MS patients (99.5%), but it is also frequent in healthy people (90−95%) [33] and is linked to infectious mononucleosis [34]. This finding implies that an EBV infection is required but not enough for the onset of MS; other viruses may cause MS relapse [35-37]. Although the role of viral infection in the etiology of T1D is unclear [38], several case reports indicate that people acquired T1D after contracting EBV and developing viral antibodies [39,40] and demonstrated that cytotoxic T-lymphocytes target β cells of the pancreas [41]. Nevertheless, there is no proof that EBV causes T1D. Multiple studies have found a significant risk of T1D among newborns whose mothers were infected with enteroviruses during pregnancy, such as the Coxsackie virus [42,43]. However, recent research has called into question the validity of this link [44,45].

Multiple sclerosis and rheumatoid arthritis

The global prevalence of rheumatoid arthritis (RA) is estimated to be 1%, while the prevalence of MS is considered to be 0.1%. RA and MS have been linked to various ADs. However, the two diseases have only been linked in a few cases. In a case-control study [46], five incidences of RA were discovered in 155 MS patients. According to another study, 15% of French MS patients had a first-degree relative with an AD (such as Grave's disease, RA, vitiligo, or T1D). Many lines of evidence suggest that MS, like RA, is a T cell-mediated AD with genetic variables having a role in pathogenesis. In terms of pathophysiology, RA and MS have a lot in common.

The major histocompatibility complex (MHC) gene plays a role in the likelihood of developing ADs [47-49]. Aside from the MHC gene, some genetic loci have lately been linked to the risk of ADs. RA, MS, T1D, and other disorders are connected to a collection of alleles in the HLA-DRB1 locus on the short arm of chromosome 6 (6p21.3) [49-52]. MHC class II β chain is encoded by this gene, which is expressed on APCs such as dendritic cells and B-cells. HLA-DRB1 alleles linked to RA, in particular, encode a "shared epitope" (SE), which is a conserved amino acid sequence of antigen-binding sites [47]. Despite the varying SE allele frequencies among ethnic groups, GWAS of RA in the 1980s and 1990s revealed a relationship at the HLA locus among different ethnic groups [47,53-55]. In the 2000s, the strongest link was seen in the MHC region in GWAS of MS [56,57]. Baranzini et al. reported the results of a GWAS published in 2009 about ADs, including RA and MS, and used a network-based analysis to present common susceptibility genes [58,59]. A substantial cluster of genes was found in the MHC region for RA and MS. This could, however, be due to the high linkage disequilibrium in this chromosomal area. In addition, RA and MS shared 12 non-MHC genes [58].

Many factors could explain the apparent link between MS and RA. Serum interleukin-17 (IL-17) levels are higher in both MS [60] and RA patients [61], which has a crucial role in the development of both diseases [60,62-65]. In addition to promoting angiogenesis [66], enhancing osteoclastogenesis, receptor activator of nuclear factor-kB ligand expression in fibroblast-like synoviocytes [67], intensifying matrix metalloproteinase-3 and IL-6 in RA cartilage progenitor cells [68], IL-17 is used to assess radiographic progression in patients with RA [69], C-reactive protein (CRP), anti-cyclic citrullinated peptide antibody, histopathological alterations [70], and treatment efficacy [71]. T-helper type 17 (Th17) cells multiply in MS [72,73] as well as RA [74]. Th17 cells, which cause MS [75-80], are also linked to RA since they generate pro-inflammatory cytokines like IL-6 and facilitate matrix metalloproteinase synthesis by synovial fibroblasts [81]. The amount of Th17 cells has a positive relationship with CRP levels, rising disease activity score [74], and diminishing treatment response [71]. Thus, one of the mechanisms through which MS enhances vulnerability to RA could be through similar immunologic pathways involving IL-17 and Th17.

Multiple sclerosis and other neurological disorders

Guillain-Barre syndrome (GBS) is a peripheral nervous system (PNS) demyelinating autoimmune illness. In contrast, MS is also a demyelinating AD that attacks the CNS. Both disorders have immunologic, genetic, and environmental pathways in common that have been hypothesized. A general inflammatory cascade elicits the pathogenesis of the disease [82]. The coexistence of the two disorders is uncommon in the research data, with only a couple of case reports mentioning it. According to many studies published as per previous literature, the presence of both diseases is not explained by chance. First of all, the association of inflammatory demyelinating polyneuropathy and MS and the occurrence of CNS demyelination after PNS demyelination in animal models [83] suggested a shared immunopathogenic background. Second, Vedeler et al. [84] established in a review article in 2001 in Norway a typical genetic relationship between MS and GBS; specific Fc-gamma receptors (FcγR) allotypes, such as Fc receptor IIA (FCRIIA) and Fc receptor IIIB (FCRIIIB), were linked to the severity of both disorders. Third, both MS and GBS can be connected to EBV, a prevalent environmental risk factor. EBV infection is one of the most important risk factors for MS and is a well-known infection that occurs before GBS [85]. In a population-based case-control study in 2010, Langer-Gould et al. found that MS patients were statistically more likely than matched controls to develop GBS later in their clinical course (p = 0.006) [83]. Furthermore, in four of the six patients in their cohort, GBS occurred before MS (Table 2). In different case report studies, only nine people with MS as well as GBS have been identified. A population-based research revealed seven patients in 2012 in Iran by Etemadifar et al. [86] (Table 2). Hassan et al. [87] described a case report in 2021 in Saudi Arabia, a 19-year-old woman who was diagnosed with GBS and then MS. However, in that case, an interesting point is the emergence of symptoms similar to GBS in the second episode, which is a unique MS presentation besides the coexistence with GBS.

Recurrent GBS was one of the most important differential diagnoses at the time, occurring in 2-5% of cases [87]. Acute development of chronic inflammatory demyelinating polyneuropathy was another option. On the other hand, the "accidental" detection of central lesions in a routine MRI and the third clinical attack favored the coexistence of the two diseases [87] (Table 2).

**Table 2:** Summary of included studies linking multiple sclerosis and Guillain-Barre syndrome. MS: multiple sclerosis; GBS: Guillain-Barre syndrome; OR: odds ratio; CI: confidence interval

References	Design	Data collected	Number of cases	Population	Results	Conclusion
Hassan et al. (2021) [87]	Case report		1	Nineteen-year-old female in Saudi Arabia	Development of MS after GBS	Co-occurrence of both GBS and MS may be present
Etimadifar et al. (2012) [86]	Retrospective population-based survey	Isfahan MS society (IMMS) records from 04/2003 to 07/2010	3,522 MS patients (2,716 women, 806 men)	Adult MS patients in Iran	Among MS patients: seven patients (six females and one male) were diagnosed with GBS before	Development of MS in individuals with a history of GBS is more than a simple incidental event
Langer-Gould et al. (2010) [83]	Case-control	Northern California, Kaiser Permanente Medical Care program	5,296 MS patients, 26,478 controls	Adults with and without MS in California	Among adult MS cases were diagnosed with GBS: OR = 5.0; (95% CI: 1.6-15.4)	MS patients are more likely to develop GBS-MS may share environmental, genetic, and other Immune alterations with GBS

**Figure 2:**
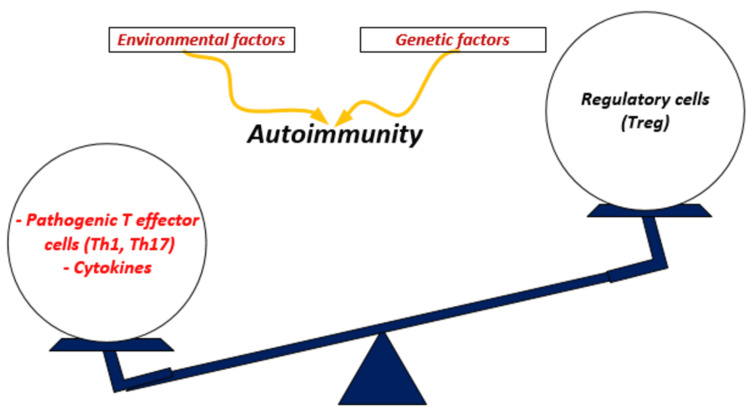
A potential link between genetic and environmental triggers with autoimmunity. Th1: T-helper type 1; Th17: T-helper type 17. Image credits: Zineb Barkhane.

The coexistence of myasthenia gravis (MG) and MS is more than just a coincidence, and the link may be undetected due to possible overlap of pathology, particularly bulbar and ocular symptoms. The pathophysiology of MS and MG has many commonalities. Both MS and MG are thought to be primarily caused by T cells [88,89]. Increased numbers of Th1 and Th17 cells and their related cytokines IL-1, IL-6, IL-17, interferons (IFN), and tumor necrosis factor (TNF) are found in MS and MG patients [89-94]. Furthermore, these patients' T regulatory (Tregs) cells exhibit several recognized abnormalities. Tregs are cells that reduce the effector CD4^+^ T cells (Teff) that cause autoimmune reactions (Figure 2). As detailed in 2017 by Danikowski et al., Tregs dysfunction and incapacity to repress Teff cells to keep self-tolerance are implicated in various ADs; Tregs suppressive capacities have also been found to be reduced in MS and MG patients [95]. Tregs augmentation therapies have been used as a treatment option for these patients, which boost Tregs-dependent B cell death or suppression, particularly in MG, by increasing Tregs repressive function and/or numbers, increasing Tregs migration, or enforcing tolerogenic signals from Tregs [95]. More research and clinical studies are required before being used in clinical practice. Also, Oksenberg et al. in 1990 in the USA described the function of T-cells and predisposition in MS and MG, claiming that because the T-cell receptor (TcR), which is specific for the antigen, is important for the immune response, mutations in the gene encoding this receptor could have a role in the development and inheritance of these ADs [96]. They demonstrate that the TcR alpha chain polymorphisms are linked to MS and MG susceptibility, raising the probability of developing haplotype-specific immunotherapeutic monoclonal antibodies that could suppress the autoimmune T-cell response [96]. In addition, Lu et al., in 2013, described the role of B cells, plasma cells, and self-reactive antibodies in MG and MS pathogenesis [97]. The oligoclonal antibody in the CSF is a specific diagnostic finding in MS [97]. Rituximab, an anti-cluster of differentiation 20 (CD20) monoclonal antibody that suppress B cells, is a strong immunomodulatory treatment for MS individuals, supporting this theory [97]. The findings show that humoral immune-mediated pathogenesis may play a role in MS etiology.

Although MS and MG are two different ADs, some research suggests a link [98]. According to a retrospective analysis [98], five out of 1,718 MS patients (0.29%; expected percentage (0.005-0.015%)) had MG. In another study [99], MS was found to precede six to eight years of MG in five out of eight participants. The presentation of MG can occur prior to or following the development of MS, and the period of onset can be variable from one person to another. Still, a few cases of primary progressive MS or MG crisis were described in a case report study by Gharagozli et al. in 2011 in Iran [100].

Multiple sclerosis and other autoimmune diseases

MS has been found to coexist with other ADs in both individuals (poly-autoimmunity) and their families (intrafamilial autoimmunity), implying that MS has many pathological features in common with ADs [101-106]. Even though there is possible common pathogenesis, the exact cause of ADs is still unknown; multifactorial environmental triggers and immunological and genetic vulnerability have all been implicated. 

Autoimmunity is caused by a breakdown of central and peripheral tolerance, immune cells, pro-inflammatory cytokines, transcription factors, Treg cells, and end-organ tissues all play an essential role in the onset and progression of autoimmunity [107]. In combination with apoptotic processes, chronic inflammation and pro-inflammatory cytokine release may be a possible route for generating the chronic dysfunction seen in MS and other ADs.

MS and other ADs, such as autoimmune thyroid disease (AITD), T1D, inflammatory bowel diseases (IBD), dermatological ADs (psoriasis, vitiligo), scleroderma, systemic lupus erythematosus (SLE), RA, ankylosing spondylitis (AS), idiopathic thrombocytopenic purpura (ITP), MG, and autoimmune hepatitis, all confirm that injury is caused by the immune response [108-112]. Even though GWAS has found links between many small nucleotide polymorphisms (SNPs) and AD, the involvement of some genes in a given locus can only be hypothesized [113,114]. Individuals who suffer from an AD are more likely to have another organ-specific AD.

Also, many environmental factors, such as lack of exposure to sunlight and multiple common infections (EBV and *Helicobacter pylori*) may damage the cell, leading to autoantigen exposure and epitope mimicry, as well as activation of APCs. At this point, *H. pylori* infection could represent an environmental factor that causes poly-autoimmunity in MS patients [115-118]. Sjogren's syndrome, systemic sclerosis, autoimmune pancreatitis, autoimmune hepatitis, primary biliary cirrhosis, primary sclerosing cholangitis, or hepatitis C virus-related liver disease are all linked to *H. pylori* infection as well as MS pathogenesis [119-121].

Deretzi et al. performed a cohort study in 2015 in Greece. The prevalence of poly-autoimmunity in 2,140 MS patients (female to male ratio: 2.1:1) was 8.3% (vs 6.07% in 1580 matched controls (p = 0.008) [122]. The prevalence of multiple autoimmune syndrome (MAS) was 1.0%. Organ-specific ADs were the most common illnesses seen in MS patients. Between female and male MS patients, there was no statistical difference in total rates of ADs [122]. Women had an increased incidence of AITD (P = 0.004), while men had an increased incidence of iritis (P = 0.039) and AS (P = 0.003). MS was diagnosed in the same year as AD in 7.4% of patients with other ADs, 42% before AD, and 50.6% after AD [122] (Table 3). Henderson et al. conducted a case-control study using a questionnaire design in Australia in 2000. The study has shown that the prevalence of ADs other than MS is higher in patients with MS and their first-degree relatives than in controls and their first-degree relatives [123] (Table 3). A case-control study performed by Karni and Abramsky in 1999 reported the increased occurrence of AITD in females with MS [124] (Table 3). Midgard et al. conducted a hospital-based interviewer questionnaire study in 1996 in Norway of hospitalized patients. They found that the combined prevalence of RA, psoriasis, and goiter was significantly higher in MS patients than in controls [46] (Table 3). Seyfert et al. performed a prospective case-control interview study in 1990 in Berlin and found a significantly increased occurrence of total ADs in MS patients compared with a control group [125] (Table 3).

**Table 3:** Summary of included studies linking multiple sclerosis and other autoimmune diseases. MS: multiple sclerosis; AD: autoimmune diseases; OR: odds ratio; CI: confidence interval.

References	Design	Data collected	Number of cases	Population	Results	Conclusion
Deretzi et al. (2015) [122]	Cross sectional control study	Between 2000 and 2011	MS patients: 2,140; controls: 1,580	Hospitalized MS patients in Northern Greece	Prevalence of poly-autoimmunity in MS patients: 8.3% vs 6.07% in controls. Prevalence of multiple autoimmune syndrome: 1%	Poly-autoimmunity occurs more frequently in MS patients
Henderson et al. (2000) [123]	Case control study	Questionnaire between 1998 and 1999	MS patients: 117; first- degree relatives of MS patients: 722; controls: 222; first-degree relatives of controls: 1582	MS patients in Australia	Prevalence of AD: higher in MS than controls OR:1.7 (95% CI: 0.9-3.2) increased to 1.9 (1-3.5) after adjusting age. Prevalence of AD: higher in first-degree relatives of MS patients than first- degree relatives of controls OR:2.2 (95%CI:1.3-3.7)	Prevalence of AD is higher in MS patients and their first-degree relatives
Karni et al. (1999) [124]	Controlled prospective study	Patients are seen over 30 months period	MS patients: 391; controls: 158	Jews in Hadassah Hebrew University Hospital	Thyroid disorders are at least three times more common in females with MS than in female controls	Thyroid disorders increased in MS patients
Midgard et al. (1996) [46]	Case-control study	Between 1976 and 1986	MS patients: 155; controls: 200	Two population-based incidences in Hordaland county Norway	Prevalence of AD: higher in MS than controls OR:2.96 (95% CI: 1.23-7.66). Prevalence of MS: higher in first-degree relatives OR:12.58 (95%CI: 1.73-552).	Higher coexistence of MS with other chronic inflammatory diseases
Seyfert et al. (1990) [125]	Prospective case-control study	Over 18 months	MS patients: 101; controls: 97	Berlin	13/101 MS patients and 2/97 controls had one or more immunologic diseases	Increased coincidence of MS with immunologic diseases

Limitations

MS has shared genetic, immunological, and environmental factors with many other ADs; this article covered the most common ADs associated with MS, such as T1D and RA. But, it does not consider MS association with the rest of ADs; future studies should assess a broader range of comorbidities. In addition, this article did not address the effects of autoimmune comorbidities on MS and the impact of MS treatments on autoimmune comorbidities, which can potentially inform choices about therapeutic regimens; more research in this area is greatly needed.

## Conclusions

The studies reviewed in this article show that MS co-occurs with other ADs because of their common physiopathology, implying genetic, immunological, and environmental factors. In summary, the clinical implication of this review article is to establish a strong link between MS and these ADs that will help the expansion and simplification of the genomic markers combined with immunological markers, both making early screening for ADs in MS patients. It will then be possible to distinguish multiple genes that control the onset and progression of MS and other ADs in their interaction with environmental factors. This will be important for understanding the underlying disease mechanisms and open the way to new therapeutic approaches and prediction markers that allow earlier immune intervention. This is why we feel that the association between MS and other ADs needs more in-depth research studies to construct a more organized and direct approach to early screening and managing these conditions.
